# Ce6-GFFY is a novel photosensitizer for colorectal cancer therapy

**DOI:** 10.1016/j.gendis.2024.101441

**Published:** 2024-10-28

**Authors:** Wei Qiao, Shuxin Li, Linna Luo, Meiling Chen, Xiaobin Zheng, Jiacong Ye, Zhaohui Liang, Qiaoli Wang, Ting Hu, Ling Zhou, Jing Wang, Xiaosong Ge, Guokai Feng, Fang Hu, Rongbin Liu, Jianjun Li, Jie Yang

**Affiliations:** a Department of Endoscopy, State Key Laboratory of Oncology in South China, Guangdong Provincial Clinical Research Center for Cancer, Sun Yat-sen University Cancer Center, Guangzhou, Guangdong 510060, China; b State Key Laboratory of Oncology in South China, Guangdong Provincial Clinical Research Center for Cancer, Sun Yat-sen University Cancer Center, Guangzhou, Guangdong 510060, China; c Department of Nuclear Medicine, State Key Laboratory of Oncology in South China, Guangdong Provincial Clinical Research Center for Cancer, Sun Yat-sen University Cancer Center, Guangzhou, Guangdong 510060, China; d Department of Oncology, Affiliated Hospital of Jiangnan University, Wuxi, Jiangsu 214062, China; e Guangdong Provincial Key Laboratory of Construction and Detection in Tissue Engineering, Biomaterials Research Center, School of Biomedical Engineering, Southern Medical University, Guangzhou, Guangdong 510515, China; f Department of Ultrasound, Sun Yat-sen Memorial Hospital, Sun Yat-sen University, Guangzhou, Guangdong 510120, China; g Guangdong Provincial Key Laboratory of Malignant Tumor Epigenetics and Gene Regulation, Sun Yat-sen Memorial Hospital, Sun Yat-Sen University, Guangzhou, Guangdong 510120, China

**Keywords:** Anti-tumor immunity, Ce6-GFFY, Colorectal cancer, Novel photosensitizer, Photodynamic therapy

## Abstract

Photodynamic therapy is an “old” strategy for cancer therapy featuring clinical safety and rapid working, but suitable photosensitizers for colorectal cancer therapy remain lacking. This study synthesized a novel photosensitizer termed Ce6-GFFY based on a self-assembling peptide GFFY and a photo-responsive molecule chlorin e6 (Ce6). Ce6-GFFY forms macroparticles with a diameter of ∼160 nm and possesses a half-life of 10 h, as well as an ideal tumor-targeting ability in mouse models. Ce6-GFFY effectively penetrates cells and generates numerous reactive oxygen species upon 660 nm laser irradiation. The reactive oxygen species promotes the accumulation of cytotoxic T cells and decrease of myeloid-derived suppressor cells in the tumor microenvironment through immunogenic cell death, thus prohibiting the growth of both primary and metastatic tumors after once treatment. This study not only provides a strategy for photosensitizer development but also confirms a promising application of Ce6-GFFY for colorectal cancer therapy.

## Introduction

Colorectal cancer (CRC) is one of the most malignant diseases that easily metastasizes to important organs such as the liver, lung, and ovary.^1^ Many strategies have been employed for clinical CRC therapy such as chemotherapy, targeted therapy, and immunotherapy.^2^, ^3^, ^4^ Although chemotherapy is still the preferred strategy for CRC treatment, most patients only show a good response at the first treatment, and long-term administration is always not effective in reducing tumor recurrence.^5^ Besides, the well-known acute toxicity also confines the application of chemotherapy in some patients.^6^ The emergence of targeted drugs such as cetuximab has greatly reduced drug toxicity and improved the effectiveness of CRC therapy, but the frequent gene mutations such as *P53* and *KRAS* in CRC cells always lead to clinical resistance to targeted drugs.^7^^,^^8^ Immune checkpoint therapy has brought a breakthrough in cancer therapy, and the programmed cell death 1 (PD1) antibody has been approved for the treatment of CRCs with high levels of microsatellite instability.^3^ However, CRCs with low levels of microsatellite instability exhibit a conventional morphology with minimal tumor-infiltrating lymphocytes, resulting in limited response rates among patients.^9^ Therefore, there is an urgent need for the development of novel anti-tumor drugs that can effectively treat CRC patients with different gene statuses.

In contrast to traditional targeted drugs, photodynamic therapy (PDT) destroys cancer cells immediately without considering their genetic status, making it a promising approach for treating various types of CRC.^10^ PDT contains two individual non-toxic components, namely a laser device and a photosensitizer,^11^ among which the laser device is used to generate a specific laser with an appropriate wavelength to activate the photosensitizer,^12^ and the photosensitizer is used to target tumors and generates reactive oxygen species (ROS), especially singlet oxygen (^1^O_2_) upon laser irradiation to induce cell death.^13^ PDT-induced cell death always promotes the release of tumor-associated antigens into the microenvironment, these released tumor-associated antigens can be presented by antigen-presenting cells to activate cytotoxic T cells for specific tumor killing.^14^ Besides, several damage-associated molecular patterns related to immunogenic cell death (ICD) have been demonstrated, including the release of large amounts of ATP and high-mobility group box 1 (HMGB1) into the extracellular milieu, and the translocation of calreticulin (CRT) from the endoplasmic reticulum to the cell surface.^15^^,^^16^

Despite being approved for clinical use 30 years ago, PDT is not widely utilized in cancer therapy due to the lack of suitable photosensitizers. Although first-generation photosensitizers such as hematoporphyrin and their derivatives show excellent photophysical and electrochemical properties, the self-quenching behavior, complex composition, and poor photochemical stability significantly impede their practical applications.^17^^,^^18^ Second-generation photosensitizers such as chlorin E6 (Ce6) possess specific molecular structures, enhanced tumor-targeting capabilities, increased ROS production, and augmented cell-killing potential.^19^ Ce6 is a degradation product of chlorophyll and generates numerous ROS upon the irradiation of 650–700 nm, and its derivatives such as talaporfin sodium and temoporfin have been approved for clinical cancer therapy.^20^^,^^21^

The third-generation photosensitizers feature the highest targeting ability and suitable half-lives achieved through the conjugation of second-generation photosensitizers with targeting molecules such as antibodies, peptides, or nanoparticles.^22^ Polypeptide is a kind of natural material with protein homology, good biocompatibility, and low toxicity. As a representative of the new generation of biological materials, polypeptide structure is simple and easy to synthesize, indicating a potential use in encapsulating and transporting small molecule drugs.^23^^,^^24^ Gly-Phe-Phe-Tyr (GFFY) peptide was reported to possess the capability of self-assembling and was used for the development of different drugs or biomaterials. A highly sensitive aggregation-induced emission (AIE) fluorescent light-up probe TPE-GFFYK (DVEDEE-Ac) was designed based on the peptide GFFY, which can induce the ordered self-assembly of AIE luminogen (AIEgen), yielding close and tight intermolecular steric interactions to restrict the intramolecular motions of AIEgens for excellent signal output.^25^ The naphthylacetic acid-modified D-enantiomeric GFFY (D-Nap-GFFY) can form a nanofiber hydrogel which is selectively taken up by antigen-presenting cells, and D-Nap-GFFY-encapsulated T317 (D-Nap-GFFY-T317) enhances dendritic cell maturation and infiltration into tumors, increases CD3^+^/CD8^+^ cells in tumors, and inhibits tumor angiogenesis.^26^ The naphthylacetic acid-modified GFFY (Nap-GFFY) also is a novel vaccine adjuvant, antigens can be easily incorporated into the hydrogel by a vortex or by gently shaking before injection, and the vaccines can stimulate strong CD8^+^ T-cell responses, which significantly inhibits tumor growth.^27^ In addition, a naproxen acid-modified tetra peptide of GFFY (Npx-GFFY) hydrogels enhances the protection of the H7N9 vaccine and is a promising adjuvant for H7N9 vaccines against highly pathogenic H7N9 virus.^28^ Besides, a previous study confirmed that the hydrogel formed by GFFY peptides has good stability in terms of both humoral immunity and anti-tumor cellular immunity.^29^ The diameter of self-assembled particles formed by GFFY peptides varies depending on the coupling molecules used, but most macroparticles show a size of approximately 100 nm.^30^ This size ensures that the macroparticles can target and penetrate tumor tissues through the enhanced permeability and retention (EPR) effect, a well-established mechanism by which macroparticles ranging from 100 nm to 800 nm in size enter solid tumors.^31^

In this study, we have developed a third-generation photosensitizer, namely Ce6-GFFY, by combining the peptide GFFY with the Ce6 molecule. A series of experiments were conducted to investigate the functional mechanism of Ce6-GFFY in CRC therapy. Our findings indicate that Ce6-GFFY forms macroparticles, effectively targets and accumulates in tumor tissues, and induces significant ROS production in cancer cells upon the irradiation of a 660 nm laser. Additionally, Ce6-GFFY effectively inhibits the growth of both primary and metastatic tumors through the induction of ICD, demonstrating a promising application for the clinical treatment of CRC.

## Materials and methods

### Cells and reagents

HCT116 (human) and CT26 (mouse) CRC cell lines were purchased from ATCC (Rockville, MD, USA) and maintained in DMEM or 1640 medium at 37 °C in 5% CO_2_. The medium was supplemented with 10% fetal bovine serum (FBS; Thermo Fisher Scientific, Waltham, MA, USA). All cell lines were authenticated by short tandem repeat profiling and were tested for mycoplasma contamination. GFFY was synthesized by Synpeptide (Shanghai, China), chlorin e6 (#C829662) was purchased from Macklin Biochemical (Shanghai, China), 4′,6-diamidino-2-phenylindole (DAPI, #C1002) and 2′,7′-dichlorodihydrofluorescein diacetate (DCFH-DA, #S0033S) were purchased from Beyotime Biotechnology (Shanghai, China), propidium iodide and annexin V-FITC apoptosis detection kit (#BMS500FI-300) was purchased from Thermo Fisher Scientific (Waltham, MA, USA), and rabbit anti-CRT antibody (#ab92516) was purchased from Abcam (Boston, MA).

### Characterization of Ce6-GFFY

Ce6-GFFY was synthesized through a dehydration condensation reaction between the carboxyl of Ce6 and the amino group of GFFY, and purified using high-performance liquid chromatography to obtain the compound binding to only one GFFY peptide, then the compound was further identified using mass spectrometry. Ce6-GFFY was suspended in phosphate-buffered saline (PBS) (0.1 mg/mL) at room temperature for 10 min, then the particle size, zeta potential, and polydispersity index were measured by dynamic light scattering according to the manufacturer’s protocol. The morphological feature of Ce6-GFFY was examined using transmission electron microscopy (Tecnai Spirit T12) according to the manufacturer’s protocol. For stability examination, Ce6-GFFY macroparticles were incubated at 37 °C for 24 h, 48 h, 72 h, and 7 d, respectively, then the particle size and polydispersity index were detected by dynamic light scattering. Besides, Ce6-GFFY macroparticles were frozen at −80 °C for 1 h, and thawed at 37 °C, and then the particle size was examined by dynamic light scattering.

### CCK-8 assay

Cell viability was measured using a CCK-8 cell counting kit (Beyotime Biotechnology, Shanghai, China). 7000 cells were seeded in 96-well plates and treated with drugs at various concentrations for 1 h and then treated with or without laser irradiation for 1 min (660 nm, 0.02 W/cm^2^), followed by incubation at 37 °C for 24 h. After the addition of the CCK-8 reagent, the cells were continually incubated at 37 °C for 3 h before being detected by a microplate reader (TECAN, Victoria, Austria).

### Flow cytometry analysis

For cell endocytosis detection, CT26 and HCT116 cells were treated with Ce6 molecules (5 μM) or Ce6-GFFY (5 μM) at 37 °C for 1 h. For ROS detection, cells were treated with Ce6 molecules (5 μM), GFFY peptide (5 μM), or Ce6-GFFY (5 μM) at 37 °C for 1 h and then treated with or without laser irradiation for 1 min (660 nm, 0.02 W/cm^2^). Afterward, the cells were collected and stained with DCFH-DA (1:5000) at 37 °C for 20 min. For cell death analysis, cells were treated with Ce6 molecules, GFFY peptide, or Ce6-GFFY (CT26, 10 μM; HCT116, 5 μM) at 37 °C for 1 h and then treated with or without laser irradiation for 1 min (660 nm, 0.02 W/cm^2^). After incubation at 37 °C for 24 h, the cells were collected and stained with propidium iodide (1:100) and annexin V-FITC (1:200). For examination of CRT expression, cells were treated with Ce6 molecules, GFFY peptide, or Ce6-GFFY (CT26, 10 μM; HCT116, 5 μM) at 37 °C for 1 h and then treated with or without laser irradiation for 1 min (660 nm, 0.02 W/cm^2^). After incubation at 37 °C for 8 h, the cells were collected and blocked with 5% bull serum albumin for 10 min and then incubated with a primary anti-calreticulin antibody (#ab92516, Abcam), followed by the incubation of an Alexa Fluor 488-conjugated secondary antibody (#406416, BioLegend).

For tissues, primary and metastatic tumors were digested into single cells using the KeyGEN tissue dissociation kit (#KGA829, KeyGEN BioTECH) following standard protocol. Digested tumors were mashed through 40 μm filters into PBS and were centrifuged at 300 *g* and 4 °C for 5 min; the obtained cells were blocked with 5% bull serum albumin for 10 min and incubated with a surface antibody mixture at room temperature for 2 h. Antibodies against CD45 (APC-Cy7, #557659, BD Biosciences), CD3 (PE, #100206, BioLegend), CD8a (Alexa Fluor700, #100730, BioLegend), CD11b (PE, #101208, BioLegend), and Gr-1 (FITC, #108405, BioLegend) were used. The above treated cells were determined by flow cytometry (Beckman–Coulter) and analyzed by FlowJo v.10.8.1 software.

### Immunofluorescence and confocal microscopy detection

For cell endocytosis detection, CT26 and HCT116 cells were treated with Ce6 molecules (5 μM) or Ce6-GFFY (5 μM) at 37 °C for 1 h, and then the cells were fixed with 4% paraformaldehyde for 15 min and stained with DAPI (1: 1000 diluted in PBS) for 20 min at room temperature. For ROS detection, cells were treated with Ce6 molecules (5 μM), GFFY peptide (5 μM), or Ce6-GFFY (5 μM) at 37 °C for 1 h and treated with or without laser irradiation for 1 min (660 nm, 0.02 W/cm^2^); afterward, the cells were stained with DCFH-DA (1: 5000 diluted in PBS) at 37 °C for 20 min. For examination of CRT expression, cells were treated with Ce6 molecules, GFFY peptide, or Ce6-GFFY (CT26, 10 μM; HCT116, 5 μM) at 37 °C for 1 h and treated with or without laser irradiation for 1 min (660 nm, 0.02 W/cm^2^); afterward, the cells were incubated at 37 °C for 8 h. After being fixed using 4% paraformaldehyde for 10 min and blocked with 5% bull serum albumin at 4 °C overnight, the cells were incubated with a primary anti-calreticulin antibody (#ab92516, Abcam), followed by the incubation with an Alexa Fluor 488-conjugated secondary antibody (#406416, BioLegend). Then, the cells were stained with DAPI at room temperature for 10 min.

For tissues, paraffin-embedded samples were sectioned at 4 μm thickness. Antigen retrieval was performed by a pressure cooker (at 95 °C for 10 min) in citrate antigen retrieval solution (P0081, Beyotime). The sections were then blocked in PBS containing 2% goat serum albumin at room temperature for 1 h. Then, the sections were incubated in the mixture of two primary antibodies at 4 °C overnight. The following primary antibodies were used: rat anti-Gr-1 (#108401, BioLegend), mouse anti-Cytokeratin Pan (#ab7753, Abcam), and rabbit anti-CD8 (#bs-0648R, Bioss). The sections were washed with cold PBS and incubated with the mixture of two secondary antibodies raised in different species at room temperature in the dark for 2 h. The following secondary antibodies were used: Alexa Fluor 488 labeled anti-rabbit (#A11008, Life Technologies), Alexa Fluor 594 labeled anti-rat (#Life Technologies, A11007), Alexa Fluor 488 labeled anti-mouse (#A11001, Life Technologies), and Alexa Fluor 594 labeled anti-mouse (#A21203, Life Technologies). Then, sections were counter-stained with DAPI at room temperature for 10 min. The above treated samples were examined by laser confocal fluorescence microscopy (Olympus FV1000) and analyzed using Zeiss v.3.1 software.

### Tumorigenicity and imaging in mice

Four-to-six-week-old BALB/c mice and BALB/c nude mice were purchased from Guangdong Medical Laboratory Animal Center (Guangzhou, China). All mice were maintained under standard conditions and treated according to institutional guidelines for animal care. For primary tumor therapy, 2 × 10^5^ CT26 cells were suspended in a 1:1 mixture of PBS and matrigel and subcutaneously injected into the flanks of the mice. When the volume of tumors reached 70 mm^3^, the mice were randomized into treatment and control groups. The treatment groups received tail intravenous injections of GFFY (2.5 mg/kg), Ce6 (2.5 mg/kg), or Ce6-GFFY (5 mg/kg), and the control group received PBS treatment, both groups were treated with once 660 nm laser irradiation for 8 min (1 min on, 1 min off; 4 cycles) on the tumor region at a power of 0.2 W/cm^2^. Tumor volume and mouse body weight were recorded every three days, and tumor tissues were collected and weighed at the end of treatment. Main organs such as the heart, liver, spleen, lung, and kidney were collected for pathological analysis and the blood was collected for blood routine examination.

For metastatic tumor therapy, 2 × 10^5^ CT26 cells were subcutaneously injected into the right flank of the mice (primary tumor), and 1 × 10^5^ CT26 cells into the left flank (metastatic tumor). When the volume of primary tumors reached 200 mm^3^, the mice were randomized into treatment and control groups. The treatment groups received tail intravenous injections of Ce6-GFFY (5 mg/kg) and the control groups received the treatment of PBS; both groups were treated with once 660 nm laser irradiation for 8 min (1 min on, 1 min off; 4 cycles) on the primary tumor region at a power of 0.2 W/cm^2^. Tumor volumes were recorded every two days and tumor tissues were weighted and collected for further analysis such as immunofluorescence and flow cytometry detection at the end of treatment.

For *in vivo* imaging, Ce6-GFFY (5 mg/kg) and Ce6 molecules (2.5 mg/kg) were injected into mice burdened with or without xenograft tumors through the tail vein (100 μL/mouse), and the fluorescence intensity of mice or main tissues such as brain, heart, liver, spleen, lung, kidney, intestine, and stomach was detected and analyzed with an IVIS spectrum imaging system (PerkinElmer, MA, USA). All animal experiments were approved by The Institutional Animal Care and Use Committee at Sun Yat-sen Cancer Center.

### Statistical analysis

Statistical analyses were performed using GraphPad Prism 8. Experiments were performed with 3 biological replicates, and the data from three independent experiments were presented as mean ± standard deviation and were compared using an unpaired *t*-test (groups ≤2) or ordinary one-way ANOVA (groups ≥3), and data with two independent variables was analyzed using two-way ANOVA. *P* < 0.05 was considered statistically significant (∗*P* < 0.05; ∗∗*P* < 0.01; ∗∗∗*P* < 0.001, and ∗∗∗∗*P* < 0.0001).

## Results

### Synthesis and characterization of Ce6-GFFY

**Figure 1:**
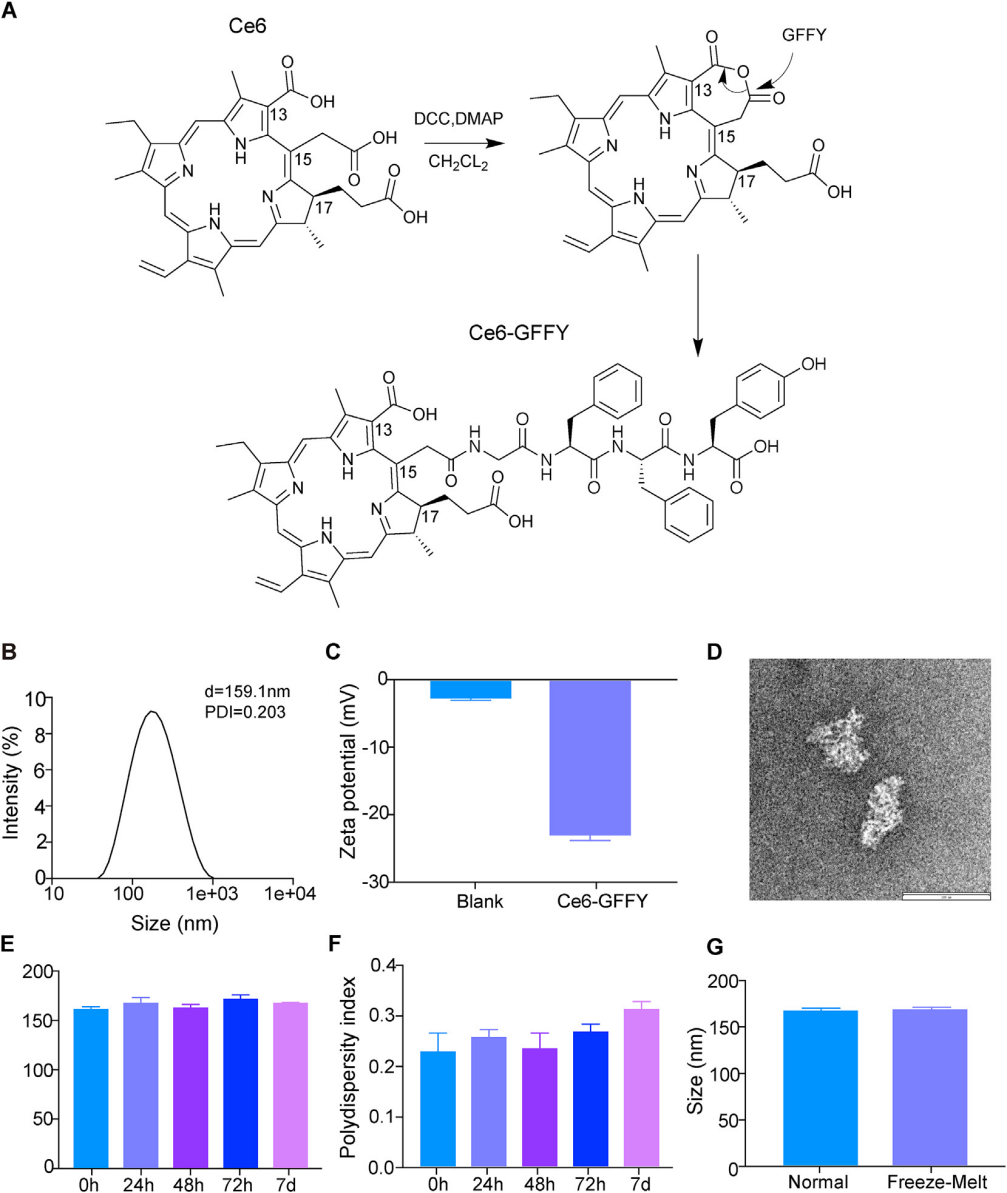
Synthesis and characterization of Ce6-GFFY. **(A)** Schematic diagram of Ce6-GFFY synthesis. DCC, N, N′-dicyclohexylcarbodiimide; DMAP, 4-dimethylaminopyridine; CH_2_CL_2_, dichloromethane. **(B)** The size distribution of Ce6-GFFY macroparticles was analyzed using DLS. d, diameter; PDI, polydispersity index; DLS, dynamic light scattering. The data are representative of five independent experiments. **(C)** The Zeta potential of Ce6-GFFY macroparticles was analyzed using DLS. Blank, PBS. The data are representative of five independent experiments. **(D)** Ce6-GFFY macroparticle image was photographed by transmission electron microscopy. Scar bar, 100 nm. **(E, F)** Particle size (E) and polydispersity index (F) of Ce6-GFFY macroparticles incubated at 37 °C at different times as indicated was detected by DLS. The data are representative of five independent experiments. **(G)** Particle size of Ce6-GFFY incubated at room temperature (normal) or underwent −80 °C/37 °C freezing-thawing (Freeze-Melt) was detected by DLS. The data are representative of five independent experiments.

Ce6-GFFY was synthesized by coupling chlorin-e6 with peptide GFFY. To ensure a relative homogeneity of the Ce6-GFFY molecules used in subsequent experiments, we further performed high-performance liquid chromatography purification after the chemical synthesis reaction and obtained a compound binding to only one GFFY peptide (Fig. S1A). Besides, the Ce6-GFFY molecules were further confirmed using mass spectrometry (Fig. S1B). We also carried out the proton nuclear magnetic resonance analysis to identify the molecular feature of Ce6-GFFY. The nuclear magnetic resonance data successfully identified the distribution of ^1^H on different functional groups, indicating that the molecular structure of Ce6-GFFY is relatively complex (Fig. S1C). However, the information provided by nuclear magnetic resonance is limited, making it difficult to determine the specific coupling site of the peptide GFFY on the Ce6 molecule. In fact, almost every photosensitizer developed based on Ce6 has encountered structural confirmation challenges. For example, talaporfin is a photosensitizer synthesized by coupling a single aspartic acid to the carboxyl group of Ce6 and has been approved for clinical use, but it was impossible to determine the coupling position of this amino acid for a long time. However, based on the chemical synthesis processes, we can make reasonable conclusions about the molecular structure of the photosensitizer. According to the previous studies, an anhydride will be firstly formed between the Ce6 15^2^ and 13^1^ carboxylic acid groups during the synthesis of Ce6-based photosensitizers, and this is more likely than a larger ring anhydride between the 17^3^ and 15^2^ acids.^32^ This phenomenon has been verified in a wide variety of nucleophiles such as ethoxide, propylamine, isopropylamine, ethanolamine, p-tolylthiolate, phenoxide, isobutoxide, and benzyloxide; all of them yield the 15^2^-conjugates, with several of these structures being confirmed by single-crystal X-ray structures.^33^ For talaporfin, the aspartic acid nitrogen atom undergoes nucleophilic attack upon the aliphatic side of the anhydride to produce the 15^2^ conjugates and the structure has also been demonstrated using single-crystal X-ray diffraction.^34^ In this study, the synthesis processes of Ce6-GFFY are the same as that of talaporfin, so the coupling position of the peptide GFFY is most likely to be on the 15^2^ carboxylic acid group of Ce6 (Fig. 1A).

Then, we examined the molecular characteristics of Ce6-GFFY from various perspectives. Dynamic light scattering analysis showed that Ce6-GFFY formed macroparticles when suspended in PBS, the average diameter of the polymers was 158.7 ± 2.8 nm (Fig. 1B), and the zeta potential was −23.1 ± 0.9 mV (Fig. 1C). We further confirmed the aggregation of Ce6-GFFY molecule using transmission electron microscopy, and the data showed that Ce6-GFFY formed irregular polymer with a uniform size distribution (Fig. 1D). Then, we explored the stability of Ce6-GFFY in different conditions. The Ce6-GFFY solution was incubated at 37 °C for different times, then the particle size and average polydispersity index were detected. Our results showed that there were almost no changes in the particle size during the incubation, even after seven days of incubation, indicating that Ce6-GFFY macroparticles had a high stability in the normal store and transport conditions (Fig. 1E, F). Moreover, the size of Ce6-GFFY macroparticles also remained stable after repeated freezing (−80 °C) and thawing (37 °C), which further identified the high stability of Ce6-GFFY macroparticles (Fig. 1G). Above all, Ce6-GFFY molecules form a uniform macroparticle aggregation in solution, and the particles remain stable under extreme conditions.

### Ce6-GFFY generates numerous ROS in CRC cells

**Figure 2:**
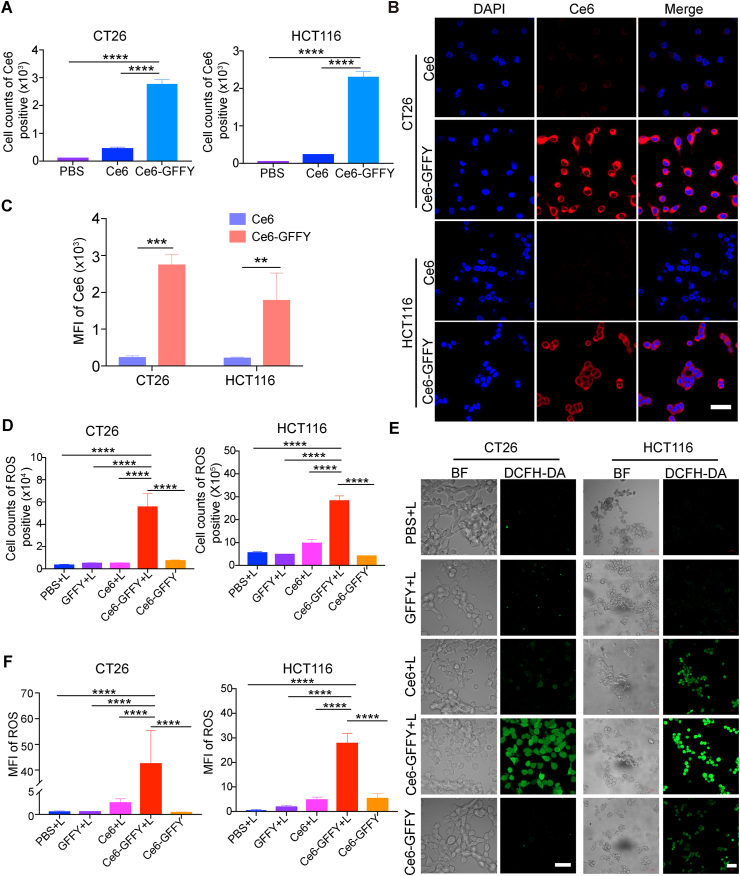
Ce6-GFFY penetrates colorectal cancer cells and generates ROS. CT26 and HCT116 cells were treated with indicated agents at 37 °C for 1 h and treated with or without 660 nm laser irradiation for 1 min at a power of 0.02 W/cm^2^. The data are representative of three independent experiments. **(A)** Cells were treated with 5 μM Ce6-GFFY or Ce6 molecules and then subjected to flow cytometry determination and the Ce6 positive cells were analyzed. **(B, C)** Cells were treated with 5 μM Ce6-GFFY or Ce6 molecules; the cellular fluorescence was determined by confocal laser scanning microscopy (B) and the mean fluorescence intensity was analyzed (C). Red, Ce6; Blue, 4′,6-diamidino-2-phenylindole (DAPI); MFI, mean fluorescence intensity. Scale bar: 40 μm. **(D)** Cells were treated with agents as indicated and then treated with or without laser irradiation; the cellular ROS levels were detected using flow cytometry assays and the ROS positive cells were analyzed. ROS, reactive oxygen species. **(E, F)** 5 μM GFFY peptide, Ce6-GFFY, Ce6 molecules, or PBS treated cells with or without laser irradiation were stained with ROS probe DCFH-DA at 37 °C for 20 min; the cellular fluorescence was determined by confocal laser scanning microscopy (E) and the mean fluorescence intensity was analyzed (F). DCFH-DA, 2′,7′-dichlorodihydrofluorescein diacetate. “L” in “PBS + L”, “GFFY + L”, “Ce6+L”, “Ce6-GFFY + L”: laser irradiation. MFI, mean fluorescence intensity; green, DCFH-DA; BF, bright field. Scale bar in CT26: 40 μm; HCT116: 50 μm. Statistical analyses were performed using one-way ANOVA except for (C), which was performed using unpaired *t*-test; bars, standard deviation; ∗∗*P* < 0.01; ∗∗∗*P* < 0.001; ∗∗∗∗*P* < 0.0001.

Successfully entering cells through endocytosis is the prerequisite for photosensitizers to exert anti-tumor effects, thus we first focused on exploring the uptake of Ce6-GFFY by CRC cells. CRC cells derived from mouse (CT26) and human (HCT116) were treated with Ce6-GFFY or Ce6 molecules, respectively. The uptake of the agents was determined through confocal laser scanning microscopy due to the specific fluorescence produced by Ce6 molecules. Flow cytometry analysis showed that the Ce6-GFFY uptake of CT26 and HCT116 cells was much higher than Ce6 molecules, which enter the cells via free diffusion (Fig. 2A). Confocal laser scanning microscopy also demonstrated that cells treated with Ce6-GFFY had a noticeable aggregation of Ce6 fluorescence in contrast to cells treated with Ce6 molecules, indicating that Ce6-GFFY has an optimal cellular endocytic activity (Fig. 2B, C).

Generally, PDT exerts its tumor cell-killing ability through ROS generated by photosensitizers under laser irradiation with specific wavelength.^35^ To confirm the ability of Ce6-GFFY to generate ROS in tumor cells, we treated CT26 and HCT116 cells with Ce6-GFFY, and subsequently monitored the intracellular ROS levels using a molecular probe, namely 2′,7′-dichlorodihydrofluorescein diacetate (DCFH-DA). Flow cytometry analysis revealed that Ce6-GFFY induced a higher level of ROS compared with the treatment with either GFFY peptides or Ce6 molecules alone upon laser irradiation, and minimal ROS generation was observed in cells treated with Ce6-GFFY without laser activation (Fig. 2D). Confocal laser scanning microscopy analysis also revealed that the Ce6-GFFY treated cells exhibited a substantial increase in ROS production upon 660 nm laser irradiation, whereas the levels of ROS were minimal in cells treated with GFFY peptides or Ce6 molecules, and negligible ROS generation was observed in non-irradiated cells (Fig. 2E, F). In summary, Ce6-GFFY macroparticles can effectively penetrate CRC cells and induce a substantial production of ROS.

### Ce6-GFFY induces immunogenic cell death

**Figure 3:**
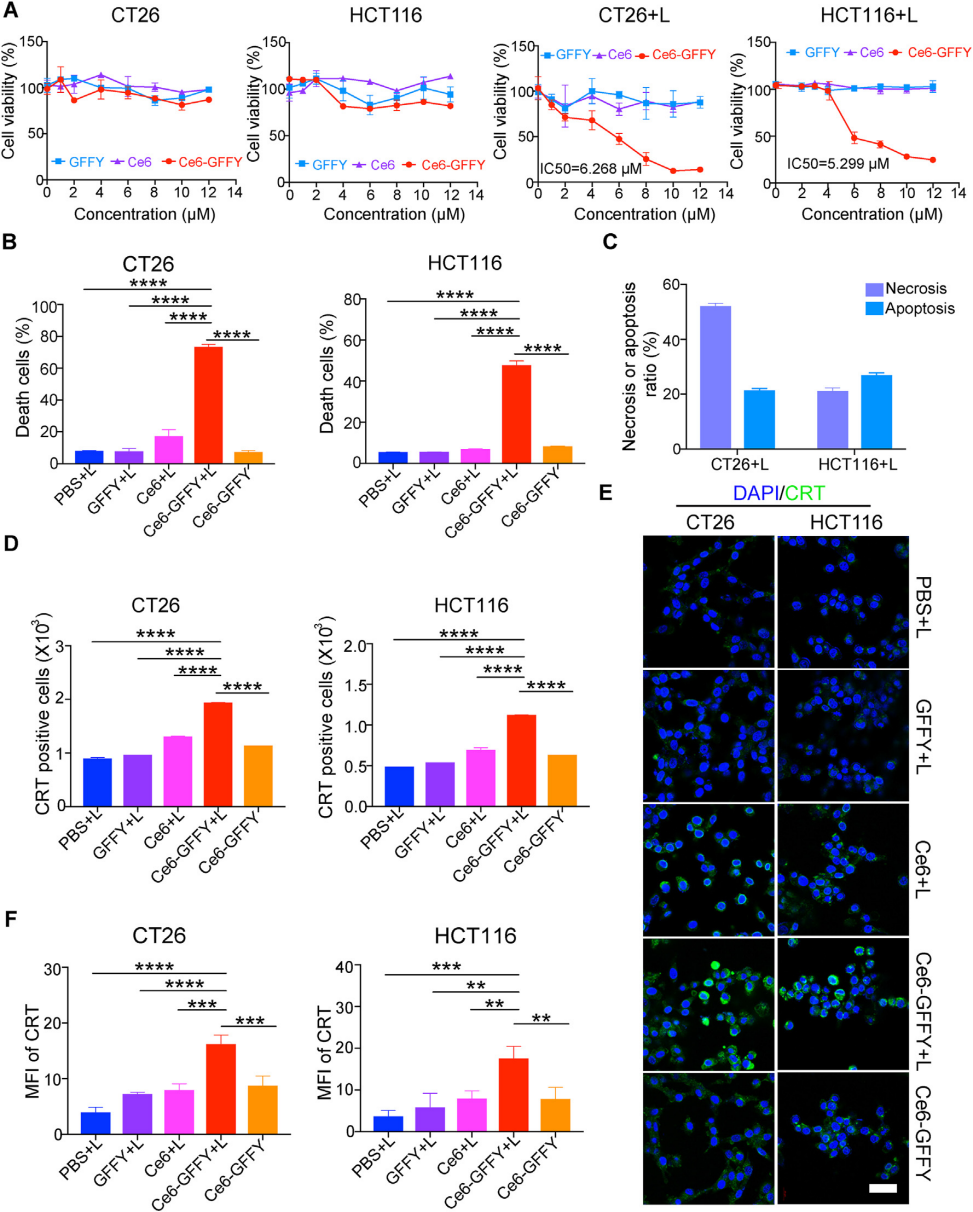
Ce6-GFFY suppresses the proliferation of colorectal cancer cells. CT26 and HCT116 cells were treated with indicated agents at 37 °C for 1 h and then treated with or without 660 nm laser irradiation for 1 min at a power of 0.02 W/cm^2^. The data are representative of three independent experiments. **(A)** Cells were incubated at 37 °C for 24 h after being treated with laser irradiation and the indicated dose of GFFY peptide, Ce6, and Ce6-GFFY, and then cell proliferation was determined using CCK-8 assays. The IC50 of Ce6-GFFY under laser irradiation was analyzed. IC50, 50 % inhibitory concentration. **(B)** Cells (CT26, 10 μM; HCT116, 5 μM) were stained with annexin V and propidium iodine dye, and then the ratio of dead cells was analyzed using flow cytometry. **(C)** The ratio of necrotic and apoptotic cells in the combined treatment of Ce6-GFFY and laser irradiation were analyzed. **(D**–**F)** After treated with the indicated agents (CT26, 10 μM; HCT116, 5 μM), cells were incubated at 37 °C for 8 h and stained with a CRT antibody, then the expression of CRT was analyzed using flow cytometry (D) and confocal laser scanning microscopy (E), and the CRT fluorescence was analyzed (F). CRT, calreticulin; green, CRT; blue, DAPI; MFI, mean fluorescence intensity. Scale bar, 40 μm “L” in “CT26+L”, “HCT116+L”, “PBS + L”, “GFFY + L”, “Ce6+L”, “Ce6-GFFY + L”: laser irradiation. Statistical analyses were performed using one-way ANOVA; bars, standard deviation; ∗∗*P* < 0.01; ∗∗∗*P* < 0.001; ∗∗∗∗*P* < 0.0001.

The cellular metabolism of ROS is tightly regulated, and excessive ROS production within a short time can result in cellular dysfunction and eventual cell death. To confirm the efficacy of ROS generated by Ce6-GFFY in suppressing CRC cells, we exposed CT26 and HCT116 cells treated with Ce6-GFFY to brief laser irradiation and assessed their proliferation status. Our results demonstrated that the ROS generated by a low concentration of Ce6-GFFY upon laser irradiation is sufficient to significantly impede the proliferation of CT26 (IC50 = 6.268 μM) and HCT116 (IC50 = 5.299 μM) cells (Fig. 3A).

Next, we investigated the mechanisms underlying the inhibitory effects of Ce6-GFFY on tumor cell proliferation. Flow cytometry analysis was carried out using propidium iodide and annexin V staining to explore the status of Ce6-GFFY treated CT26 and HCT116 cells after brief laser irradiation. The results demonstrated that the combined use of Ce6-GFFY and laser irradiation induced a mortality rate of 73% in CT26 cells and 48% in HCT116 cells, while most cells in other groups remained viable (Fig. 3B). Further analysis revealed that part of the dead cells derived from the treatment of Ce6-GFFY and laser irradiation were necrotic (52% in CT26 cells and 21% in HCT116 cells) and apoptotic (21% in CT26 cells and 27% in HCT116 cells) (Fig. 3C).

ROS-induced cell death always induces the alteration of damage-associated molecular patterns, which plays an important role in ICD. Damage-associated molecular patterns can be detected by hallmarks such as HMGB1, ATP, and surface-exposed CRT,^15^^,^^36^ among which CRT is a classical hallmark that acts as an “eat-me” signal to stimulate dendritic cells maturation and promote T cell-mediated antitumor immunity.^37^^,^^38^ Therefore, we further examined the expression of CRT in CRC cells induced by Ce6-GFFY. CT26 and HCT116 cells were treated with the combination of laser irradiation and Ce6-GFFY or other agents, and then the expression of CRT was evaluated through flow cytometry analysis using a CRT antibody. The results showed that the expression of CRT in Ce6-GFFY treated CT26 and HCT116 cells was much higher than other groups (Fig. 3D). Moreover, the immunofluorescence assays performed using confocal laser scanning microscopy further demonstrated that the CRT expression in Ce6-GFFY group was significantly up-regulated in both cells, while no significant changes were observed in other groups (Fig. 3E, F). These findings suggest that Ce6-GFFY can effectively induce ICD in CRC cells. In general, the combination of Ce6-GFFY and laser irradiation induces ICD in CRC cells, indicating a promising application of Ce6-GFFY for CRC therapy.

### Ce6-GFFY shows typical kinetics of macroparticles and an ideal tumor-targeting ability

**Figure 4:**
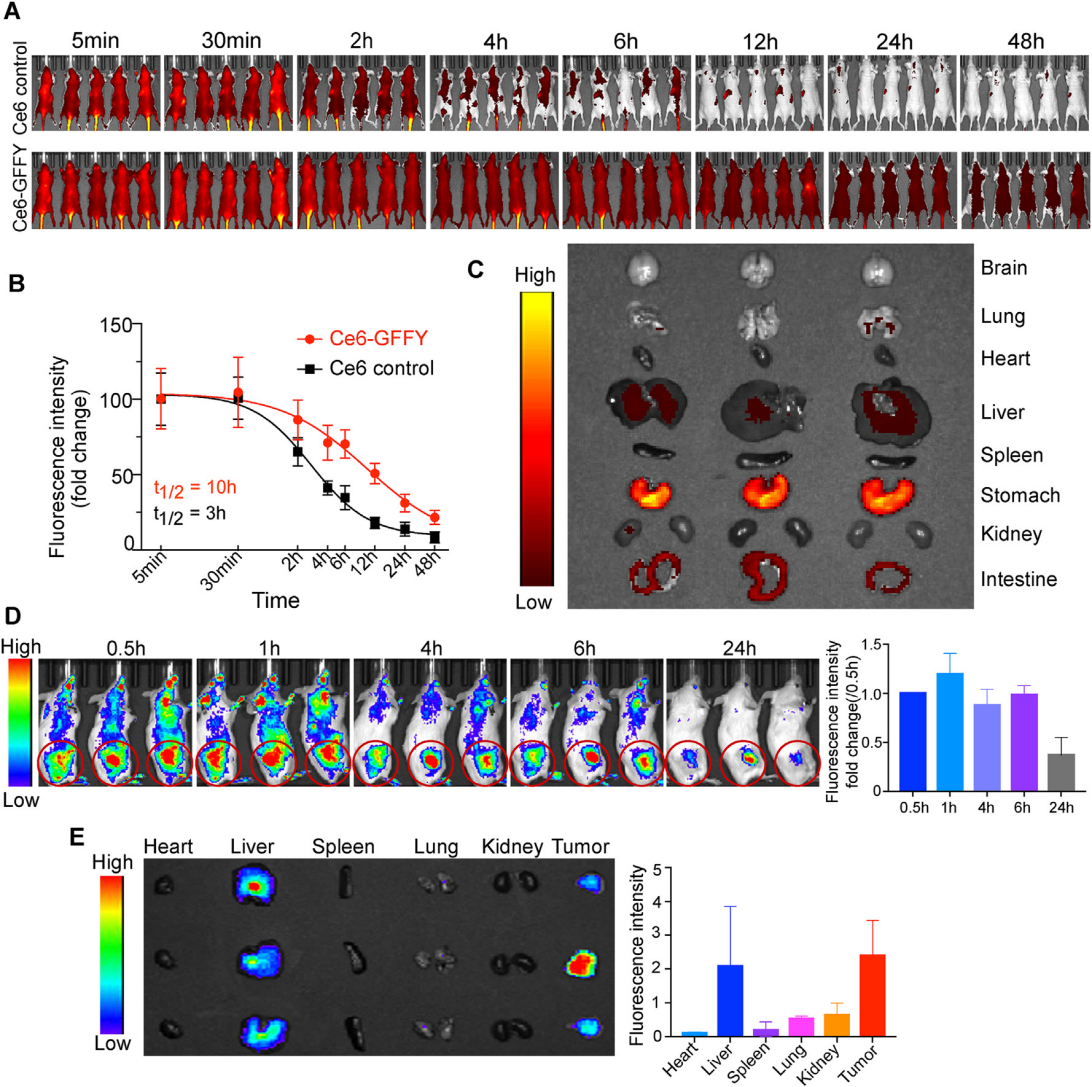
Pharmacokinetics and tumor-targeting ability of Ce6-GFFY. **(A)** Mice were treated with 2.5 mg/kg Ce6 control or 5 mg/kg Ce6-GFFY through tail vein injection, and the Ce6 luminescence was detected at the indicated time after the injection. *n* = 5. **(B)** The half-life of Ce6-GFFY and Ce6 molecules was analyzed based on the Ce6 luminescence changes. T_1/2_, half-live. **(C)** Main organs from mice in (A) (12 h) were collected for imaging. *n* = 3. **(D)** 2.5 mg/kg Ce6 control and 5 mg/kg Ce6-GFFY were injected into mice bearing CT26-derived tumors through tail vein, and the Ce6 fluorescence intensity was measured and analyzed at the indicated time after the injection (*n* = 3). Red cycle, tumor region. **(E)** Main organs, along with the tumors from mice in (D) (24 h) were collected for imaging and analysis. *n* = 3.

Ce6-GFFY induced ICD indicates that Ce6-GFFY could be a potent anti-tumor drug candidate, we thus explored its metabolism and tumor-targeting ability in mice before investigating its potential therapeutic effect. The kinetics of Ce6-GFFY metabolism were determined in mice through tail vein injection. Living imaging analysis revealed that Ce6-GFFY exhibited a prolonged retention time in the mice for over 48 h, while Ce6 control molecules were almost cleared within 12 h after injection (Fig. 4A). The Ce6 fluorescence statistics indicate that the half-life of Ce6-GFFY was 10 h in mice, whereas that of Ce6 molecules was only 3 h, indicating that the macroparticles formed by Ce6-GFFY effectively extended the retention time of the drug *in vivo* (Fig. 4B). We also collected the main organs of mice treated with Ce6-GFFY for further imaging analysis and found that the Ce6-GFFY accumulation mainly occurred in the liver, stomach, and intestine, showing a typical metabolism process of protein drugs (Fig. 4C). Then, we explored the tumor-targeting ability of Ce6-GFFY in mice bearing CT26-derived tumors. Living imaging analysis showed that Ce6-GFFY accumulated rapidly in the tumor regions after tail vein injection (Fig. 4D). Remarkably, Ce6-GFFY exhibited stable aggregation in tumor tissues even after 24 h of administration, whereas it was almost entirely cleared from normal tissues except for the liver, which serves as a metabolic organ for large particles (Fig. 4E). In summary, previous studies and our data both demonstrate that macroparticles exhibit a prolonged half-life *in vivo*, thus enhancing the drug uptake by tumors and extending the therapeutic window of drugs.^39^^,^^40^ Besides, the tumor targeting ability of Ce6-GFFY indicates that it is a promising agent for clinical CRC therapy.

### Ce6-GFFY suppresses CRC growth with minimal toxicity

**Figure 5:**
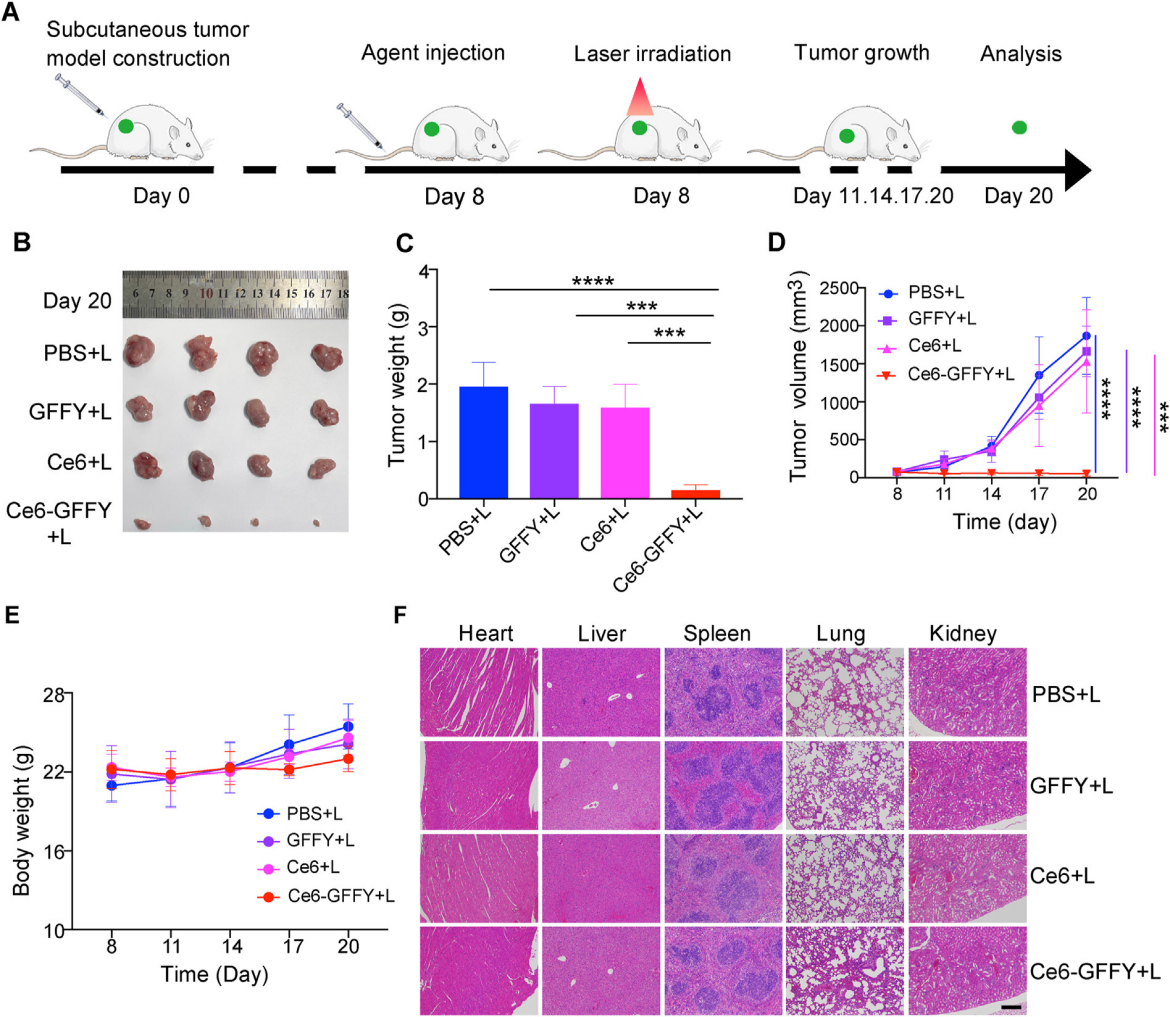
Ce6-GFFY prohibits colorectal cancer growth and has little side effects. Agents were injected through the tail vein of the CT26-derived subcutaneous tumor mice model, and the 660 nm, 0.2 W/cm^2^ laser irradiation (1 min on, 1 min off; 4 cycles) was performed 6 h after the injection. Only a single dose was administered during the entire treatment cycle. **(A)** Schematic diagram of the PDT strategy. PDT, photodynamic therapy. **(B**–**D)** Mice were treated with PBS, GFFY (2.5 mg/kg), Ce6 (2.5 mg/kg), or Ce6-GFFY (5 mg/kg), and tumor tissues were collected (B) and weighed (C) after treatment, and tumor growth curve was analyzed during treatment (D). *n* = 4. **(E)** Mice body weight was analyzed during treatment. *n* = 4. **(F)** Pathological analysis of hearts, livers, spleens, lungs, and kidneys derived from the indicated agents treated mice using hematoxylin-eosin (H&E) staining. *n* = 4. “L” in “PBS + L”, “GFFY + L”, “Ce6+L”, “Ce6-GFFY + L”: laser irradiation. Scale bar, 200 μm. Statistical analyses were performed using two-way ANOVA; bars, standard deviation; n.s., not significant; ∗∗∗*P* < 0.001; ∗∗∗∗*P* < 0.0001.

Considering the significant advantages of Ce6-GFFY in terms of metabolism and tumor targeting, we subsequently investigated its potential anti-tumor activity. A subcutaneous tumor mouse model was established using CT26 cells, and the mice were treated with Ce6-GFFY (5 mg/kg) once, followed by 8 min of laser irradiation 6 h after injection; tumor growth was assessed every three days (Fig. 5A). The data demonstrated that the combination of Ce6-GFFY and laser irradiation significantly inhibited tumor growth after a single treatment, while no significant change was observed in the groups treated with other agents combined with laser irradiation (Fig. 5B, C). Moreover, tumor growth curve statistic also confirmed the potent inhibition of tumor growth induced by the combined use of Ce6-GFFY and laser irradiation (Fig. 5D). Importantly, there was no decrease in mice weight during the treatment, indicating a minimal side effect (Fig. 5E).

We further evaluated the toxicity of Ce6-GFFY in mice using pathologic analysis. At the end of the treatment, we performed the murine blood routine analysis, data showed that the combined administration of Ce6-GFFY and laser irradiation did not elicit any significant inflammatory responses (Fig. S2A). Besides, the molecular indices indicated that Ce6-GFFY did not exert an impact on the hepatic and renal function of mice (Fig. S2B). Histopathological analysis of organs such as heart, liver, spleen, lung, and kidney of Ce6-GFFY and laser irradiation co-treated mice showed that there was no apparent toxicity in mice (Fig. 5F). Therefore, our data demonstrate that the combined use of Ce6-GFFY and laser irradiation can effectively suppress CRC growth through a single treatment with no obvious side effects, indicating that Ce6-GFFY has good drug properties.

### Ce6-GFFY activates anti-tumor immunity and suppresses metastatic CRC growth

**Figure 6:**
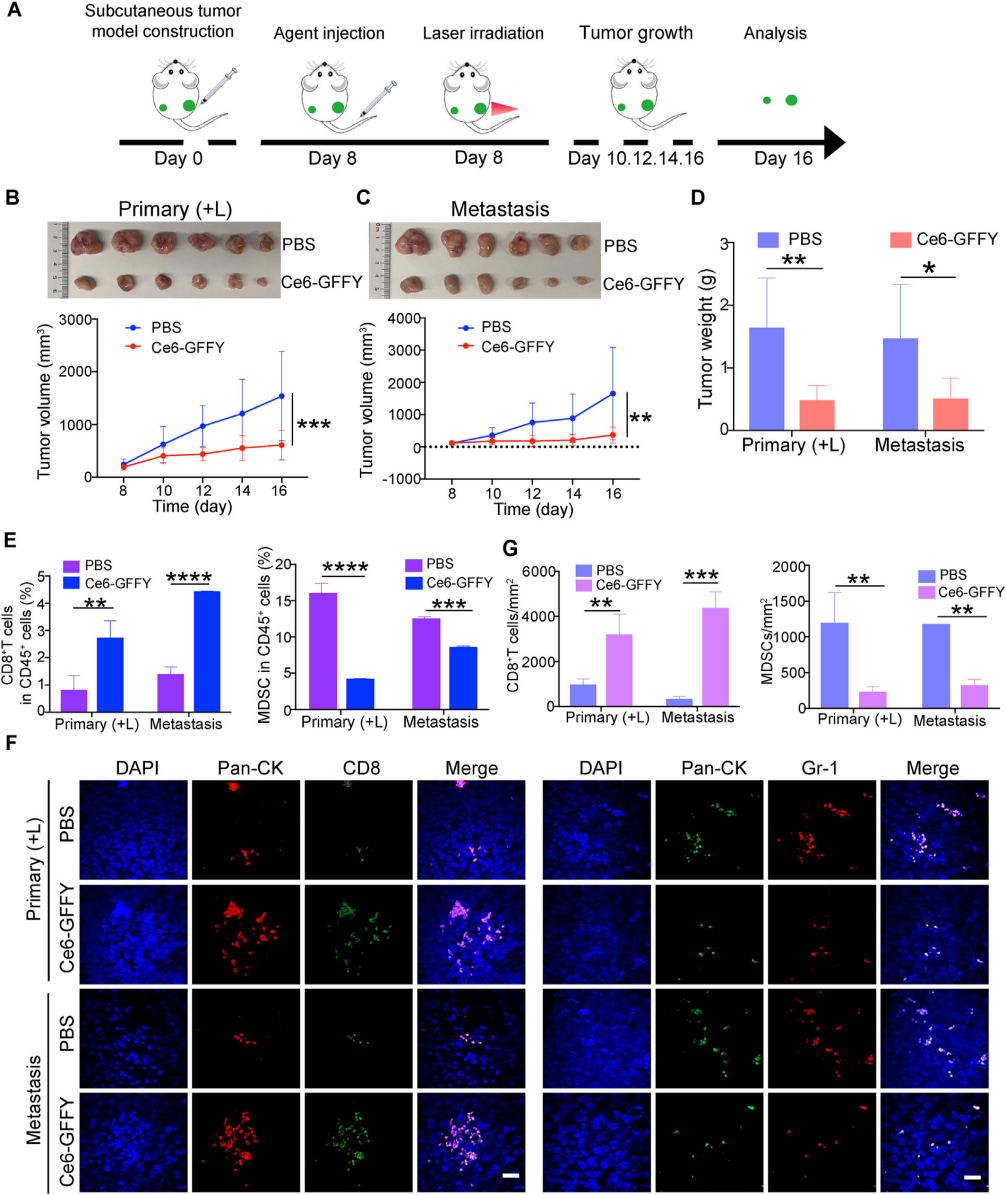
Ce6-GFFY activates anti-tumor immunity and suppresses metastatic tumor growth. Primary and metastasis tumor model was constructed by subcutaneously transplanting CT26 cells in the left (metastasis tumor) and right flanks (primary tumor) of BALB/c mouse. Then Ce6-GFFY (5 mg/kg) was injected through the tail vein of the mouse, and the 660 nm, 0.2 W/cm^2^ laser irradiation (1 min on, 1 min off; 4 cycles) was performed on the primary tumor 6 h after the injection. Only a single dose was administered during the entire treatment cycle. **(A)** Schematic diagram of the mouse model construction and PDT strategy. **(B)** Primary tumor tissues were collected and tumor growth was analyzed after treatment. *n* = 6. **(C)** Metastasis tumor tissues were collected and tumor growth was analyzed after treatment. *n* = 6. **(D)** Primary and metastasis tumors were weighed and analyzed. *n* = 6. **(E)** Primary and metastasis tumors were collected and dispersed into single cells for flow cytometry analysis, and the amount of cytotoxic T cells (CD45^+^CD3^+^CD8^+^) and myeloid-derived suppressor cells (MDSCs, CD45^+^CD11b^+^Gr-1^+^) were analyzed. *n* = 3. **(F, G)** IF staining for CD8^+^ T cells (CD8) and MDSCs (Gr-1) in primary and metastasis tumors (F), and the number of positive cells per mm^2^ was analyzed (G). *n* = 3. IF, immunofluorescence. “(L)” in “Primary (+L)”: laser irradiation. Scale bar, 20 μm. The data are representative of three independent experiments. Statistical analyses were performed using unpaired *t*-test; bars, standard deviation; ∗*P* < 0.05; ∗∗*P* < 0.01; ∗∗∗*P* < 0.001; ∗∗∗∗*P* < 0.0001.

Activating the anti-tumor immunity is an effective way to suppress cancer recurrence and metastasis.^41^^,^^42^ Considering that our cellular-level results demonstrated that the combination of Ce6-GFFY and laser irradiation induced significant immunogenic cell death, and *in vivo* experiments confirmed the drug ability of Ce6-GFFY. Therefore, we conducted a comprehensive investigation to determine whether the PDT of Ce6-GFFY could enhance the anti-tumor immune responses in mice. We first constructed a mouse model using CT26 cells, which were transplanted subcutaneously in the left and right flanks of the BALB/c mouse, respectively, to mimic the primary and metastasis tumors. Then, the mouse was administered with Ce6-GFFY via tail vein injection, followed by laser irradiation on the primary tumor area while the metastatic tumor remained unirradiated (Fig. 6A). The data showed that the growth of primary tumors was significantly suppressed by the combined use of Ce6-GFFY and laser irradiation (Fig. 6B). Interestingly, the growth of metastasis tumors was also inhibited, even in the absence of irradiation (Fig. 6C). Tumor weight analysis further substantiated the inhibitory effect on both primary and metastatic tumor growth, thereby suggesting a potential induction of anti-tumor immunity through photodynamic treatment mediated by Ce6-GFFY (Fig. 6D).

Cytotoxic T cells eliminate tumor cells by recognizing tumor-associated antigens, and thus their extensive infiltration into tumor microenvironment is essential for the induction of anti-tumor immunity.^43^ In addition, myeloid-derived suppressor cells exert immunosuppressive effects by producing arginase-1, inducible nitric oxide synthase, and other inhibitory substances, thereby playing an important role in reshaping the tumor immune microenvironment.^44^^,^^45^ Therefore, we subsequently focus on exploring the changes of cytotoxic T cells and myeloid-derived suppressor cells in tumors with or without photodynamic treatment using Ce6-GFFY. The flow cytometry analysis demonstrated that the number of cytotoxic T cells (CD45^+^CD3^+^CD8^+^) was increased and myeloid-derived suppressor cells (CD45^+^CD11b^+^Gr-1^+^) were decreased in both primary and metastasis tumors, despite only the primary tumor being subjected to laser irradiation (Fig. 6E). Moreover, immunofluorescence assays further confirmed that the cytotoxic T cells (CD8^+^) were accumulated whereas the number of myeloid-derived suppressor cells (Gr-1^+^) was decreased in both primary and metastasis tumors (Fig. 6F, G). Together, our results demonstrate that Ce6-GFFY is a promising agent in activating anti-tumor immunity and treating metastatic CRC.

## Discussion

Currently, early CRC is usually treated with surgical excision, and the advanced CRC is treated with chemoradiotherapy, targeted therapy or immunotherapy based on the genetic status such as the RAS/BRAF mutation, microsatellite instability/deficient mismatch repair.^46^^,^^47^ However, there are certain limitations to current therapeutic strategies, for example, only about 15 % of CRC patients had deficient mismatch repair with high levels of microsatellite instability, and the proportion of stage III and IV CRC patients is even lower at 11% and 5%, respectively,^48^ among which only 30%–50% CRC patients are responsive to immunotherapy.^9^^,^^49^^,^^50^ Therefore, targeted drugs developed in novel strategies are urgently needed. Unlike traditional targeted drugs, PDT requires a combination of drug and instrument (laser) to work.^51^ PDT consists of three necessary elements: photosensitizer, laser, and oxygen, among which photosensitizer determines the tumor-targeting ability and therapeutic effect of PDT.^52^ PDT is theoretically characterized by reduced toxicity and repaid effect compared with traditional drugs, which led to its FDA approval for clinical use 30 years ago.^11^ However, the clinical application of PDT in cancer therapy on a large scale has been limited due to the lack of safe and effective photosensitizers.

In this study, a novel photosensitizer Ce6-GFFY was synthesized through the conjugation of the photosensitive molecule Ce6 with the self-assembling peptide GFFY.^53^ Ce6-GFFY forms stable macroparticles with a diameter of approximately 160 nm in solution, and our data demonstrate that these particles possess excellent targeting ability for CRC and exhibit potent anti-cancer effects while causing minimal side effects; the novel photosensitizer Ce6-GFFY developed in this study can induce rapid and efficient ICD of tumor cells under laser irradiation, and thus the systemic anti-tumor immune response will be activated after irradiating at a specific tumor site; and the tumors at various metastatic sites will be eliminated via immune-mediated killing. Different from the current anti-tumor drugs, Ce6-GFFY kills tumor cells via a physical manner that ignores the gene status of CRC, and thus it has a great potential in CRC patients, especially those who cannot be treated with any existing therapeutics, such as clinical drug resistance.^54^

The tumor-targeting mechanism of Ce6-GFFY macroparticles remains uncertain; however, the EPR effect may be the underlying mechanism. The intervascular spaces in tumors contain pores ranging in size from 100 nm to 780 nm, which allow the infiltration of macroparticles.^55^ Previous studies have shown that the EPR effect primarily occurs in solid tumors due to their disorganized and abnormal vasculature compared with healthy tissues, along with the impaired lymphatic clearance from the tumor stroma, thus facilitating the penetration and retention of macroparticles in tumors.^31^^,^^56^ Besides, the shape, as well as the softness of macroparticles also have a potential impact on tumor accumulation through the EPR effect.^57^ Some studies have shown that the EPR effect is more potent when the surface of macroparticles distributes a negative charge.^58^ Our data demonstrated that Ce6-GFFY macroparticles have an irregular shape and a negative charge (derived from the Ce6 molecule) on the surface, indicating that Ce6-GFFY macroparticles have a good EPR effect, which makes it effective in tumor targeting and penetrating.

Photosensitizer is activated by laser, the wavelength of which is also contained in sunlight. Therefore, patients need to avoid exposure to sunlight for several days after receiving PDT, which has had a certain effect on their everyday lives.^59^ To address this issue, the half-life of photosensitizer needs to be suitable, a half-life of several hours of the photosensitizer seems to be suitable for the clinical application of PDT, as the patients can return to their normal lives within hours of the end of the treatment. In addition to the suitable half-life of photosensitizer, intra-tumoral injection is another effective way to reduce the side effects of PDT, the photosensitizer is injected into the tumor tissue through an endoscope or a drainage tube, then the optical fiber is guided to the tumor site where the drug was injected for laser irradiation.^60^ Compared with intravenous injection, the dose of photosensitizer used for intra-tumoral injection is very low, and laser irradiation is performed within minutes of the injection, thus little normal tissues would be penetrated by the drug during the treatment, as well as little side effects would emerge to patients.

Traditional PDT strategies seem to be more suitable for superficial tumors (such as skin cancer) than internal tumors.^61^ However, benefiting from the improvement of tumor-targeting ability and half-life of novel photosensitizers, the interventional PDT will play an important role in the treatment of a variety of tumors in the future. Ce6-GFFY macroparticle has an ideal tumor-targeting ability and a half-life of about 10 h in mice, indicating that Ce6-GFFY is a promising agent for CRC therapy.

## Conclusions

In this study, we developed a novel photosensitizer termed Ce6-GFFY by covalently combining a photo-responsive Ce6 molecule with GFFY peptide. Ce6-GFFY forms stable macroparticles with an average size of 160 nm in solution, and these macroparticles have an ideal tumor-targeting ability and a suitable half-life in mice. Ce6-GFFY macroparticles induce ICD through ROS when treated with 660 nm laser irradiation. The combined use of Ce6-GFFY and laser irradiation significantly activates anti-tumor immunity by promoting the infiltration of cytotoxic T cells and prohibiting the accumulation of myeloid-derived suppressor cells in tumors, thus suppressing the growth of both primary and metastatic CRCs. Our data indicate that Ce6-GFFY is a promising agent for CRC therapy with little side effects.

## Funding

This work was supported by grants from the 10.13039/501100001809National Natural Science Foundation of China (No. 82373174, 82002466, 82202907).

## Author contributions

**Wei Qiao:** Data curation, Formal analysis, Methodology, Resources, Software, Supervision, Validation, Visualization, Writing – original draft. **Shuxin Li:** Data curation, Formal analysis, Methodology, Resources, Software, Validation, Visualization. **Linna Luo:** Data curation, Formal analysis, Resources, Software, Validation. **Meiling Chen:** Data curation, Methodology, Software. **Xiaobin Zheng:** Methodology, Resources. **Jiacong Ye:** Conceptualization, Resources. **Zhaohui Liang:** Validation. **Qiaoli Wang:** Validation. **Ting Hu:** Validation. **Ling Zhou:** Resources. **Jing Wang:** Resources. **Xiaosong Ge:** Resources. **Guokai Feng:** Resources. **Fang Hu:** Resources. **Rongbin Liu:** Funding acquisition, Resources, Supervision. **Jianjun Li:** Conceptualization, Project administration, Resources, Supervision. **Jie Yang:** Conceptualization, Funding acquisition, Investigation, Project administration, Resources, Supervision, Writing – original draft, Writing – review & editing.

## Conflict of interests

The authors declared no competing interests.

## References

[1] Islam M.R., Akash S., Rahman M.M. (2022). Colon cancer and colorectal cancer: prevention and treatment by potential natural products. Chem Biol Interact.

[2] Kotani D., Oki E., Nakamura Y. (2023). Molecular residual disease and efficacy of adjuvant chemotherapy in patients with colorectal cancer. Nat Med.

[3] Ganesh K., Stadler Z.K., Cercek A. (2019). Immunotherapy in colorectal cancer: rationale, challenges and potential. Nat Rev Gastroenterol Hepatol.

[4] Xie Y.H., Chen Y.X., Fang J.Y. (2020). Comprehensive review of targeted therapy for colorectal cancer. Signal Transduct Targeted Ther.

[5] Wang H., Yang W., Qin Q. (2022). E3 ubiquitin ligase MAGI3 degrades c-Myc and acts as a predictor for chemotherapy response in colorectal cancer. Mol Cancer.

[6] Audisio R.A., Papamichael D. (2012). Treatment of colorectal cancer in older patients. Nat Rev Gastroenterol Hepatol.

[7] Chen P., Li X., Zhang R. (2020). Combinative treatment of β-elemene and cetuximab is sensitive to KRAS mutant colorectal cancer cells by inducing ferroptosis and inhibiting epithelial-mesenchymal transformation. Theranostics.

[8] Huang S., Benavente S., Armstrong E.A., Li C., Wheeler D.L., Harari P.M. (2011). p53 modulates acquired resistance to EGFR inhibitors and radiation. Cancer Res.

[9] Le D.T., Uram J.N., Wang H. (2015). PD-1 blockade in tumors with mismatch-repair deficiency. N Engl J Med.

[10] Ji B., Wei M., Yang B. (2022). Recent advances in nanomedicines for photodynamic therapy (PDT)-driven cancer immunotherapy. Theranostics.

[11] Agostinis P., Berg K., Cengel K.A. (2011). Photodynamic therapy of cancer: an update. CA Cancer J Clin.

[12] Wenzler J.S., Böcher S., Frankenberger R., Braun A. (2019). Feasibility of transgingival laser irradiation for antimicrobial photodynamic therapy. Photodiagnosis Photodyn Ther.

[13] Luo Y., Zeng Z., Shan T. (2022). Fibroblast activation protein α activatable theranostic pro-photosensitizer for accurate tumor imaging and highly-specific photodynamic therapy. Theranostics.

[14] Garg A.D., Krysko D.V., Vandenabeele P., Agostinis P. (2012). Hypericin-based photodynamic therapy induces surface exposure of damage-associated molecular patterns like HSP70 and calreticulin. Cancer Immunol Immunother.

[15] Kroemer G., Galluzzi L., Kepp O., Zitvogel L. (2013). Immunogenic cell death in cancer therapy. Annu Rev Immunol.

[16] Garg A.D., Dudek A.M., Ferreira G.B. (2013). ROS-induced autophagy in cancer cells assists in evasion from determinants of immunogenic cell death. Autophagy.

[17] Zhou T., Yin Y., Cai W. (2021). A new antibacterial nano-system based on hematoporphyrin-carboxymethyl chitosan conjugate for enhanced photostability and photodynamic activity. Carbohydr Polym.

[18] Xu D., Duan Q., Yu H., Dong W. (2023). Photodynamic therapy based on porphyrin-based metal–organic frameworks. J Mater Chem B.

[19] Bui H.T.H., Thi Pham T., Thi Thu Nguyen H. (2019). Transformation chlorophyll a of *Spirulina platensis* to chlorin e6 derivatives and several applications. Open Access Maced J Med Sci.

[20] Yano T., Minamide T., Takashima K., Nakajo K., Kadota T., Yoda Y. (2021). Clinical practice of photodynamic therapy using talaporfin sodium for esophageal cancer. J Clin Med.

[21] Chang K.C., Chiu K.C., Chen W.C. (2022). Effects of temoporfin-based photodynamic therapy on the *in vitro* antibacterial activity and biocompatibility of gelatin-hyaluronic acid cross-linked hydrogel membranes. Pharmaceutics.

[22] Mfouo-Tynga I.S., Dias L.D., Inada N.M., Kurachi C. (2021). Features of third generation photosensitizers used in anticancer photodynamic therapy: review. Photodiagnosis Photodyn Ther.

[23] Kumar A., Ma H., Zhang X. (2012). Gold nanoparticles functionalized with therapeutic and targeted peptides for cancer treatment. Biomaterials.

[24] Hao Y., Chen Y., He X. (2023). RGD peptide modified platinum nanozyme co-loaded glutathione-responsive prodrug nanoparticles for enhanced chemo-photodynamic bladder cancer therapy. Biomaterials.

[25] Han A., Wang H., Kwok R.T.K. (2016). Peptide-induced AIEgen self-assembly: a new strategy to realize highly sensitive fluorescent light-up probes. Anal Chem.

[26] Feng K., Ma C., Liu Y. (2021). Encapsulation of LXR ligand by D-Nap-GFFY hydrogel enhances anti-tumorigenic actions of LXR and removes LXR-induced lipogenesis. Theranostics.

[27] Luo Z., Wu Q., Yang C. (2017). A powerful CD8^+^ T-cell stimulating D-tetra-peptide hydrogel as a very promising vaccine adjuvant. Adv Mater.

[28] Wu X., Tang S., Wang Z. (2022). Immune enhancement by the tetra-peptide hydrogel as a promising adjuvant for an H7N9 vaccine against highly pathogenic H7N9 virus. Vaccines.

[29] Li M., Liu M., Shang Y. (2020). The substitution of a single amino acid with its enantiomer for control over the adjuvant activity of self-assembling peptides. RSC Adv.

[30] Hu Y., Wang Y., Deng J. (2022). Enzyme-instructed self-assembly of peptide-drug conjugates in tear fluids for ocular drug delivery. J Contr Release.

[31] Shinde V.R., Revi N., Murugappan S., Singh S.P., Rengan A.K. (2022). Enhanced permeability and retention effect: a key facilitator for solid tumor targeting by nanoparticles. Photodiagnosis Photodyn Ther.

[32] Hargus J.A., Fronczek F.R., Vicente M.G.H., Smith K.M. (2007). Mono-(l)-aspartylchlorin-e_6_. Photochem Photobiol.

[33] Chen H., Waruna Jinadasa R.G., Jiao L., Fronczek F.R., Nguyen A.L., Smith K.M. (2015). Chlorin e_6_ 13^1^:15^2^-anhydride: A key intermediate in conjugation reactions of chlorin e_6_. Eur J Org Chem.

[34] da Graça H.Vicente M., Smith K.M. (2023). Amino acid derivatives of chlorin-e6 — a review. Molecules.

[35] Yin X., Cheng Y., Feng Y. (2022). Phototheranostics for multifunctional treatment of cancer with fluorescence imaging. Adv Drug Deliv Rev.

[36] Fucikova J., Kepp O., Kasikova L. (2020). Detection of immunogenic cell death and its relevance for cancer therapy. Cell Death Dis.

[37] Obeid M., Panaretakis T., Joza N. (2007). Calreticulin exposure is required for the immunogenicity of γ-irradiation and UVC light-induced apoptosis. Cell Death Differ.

[38] Galluzzi L., Buqué A., Kepp O., Zitvogel L., Kroemer G. (2017). Immunogenic cell death in cancer and infectious disease. Nat Rev Immunol.

[39] He C., Hu Y., Yin L., Tang C., Yin C. (2010). Effects of particle size and surface charge on cellular uptake and biodistribution of polymeric nanoparticles. Biomaterials.

[40] Ejigah V., Owoseni O., Bataille-Backer P., Ogundipe O.D., Fisusi F.A., Adesina S.K. (2022). Approaches to improve macromolecule and nanoparticle accumulation in the tumor microenvironment by the enhanced permeability and retention effect. Polymers.

[41] Couzin-Frankel J. (2013). Breakthrough of the year 2013. Cancer immunotherapy. Science.

[42] Zhao W., Jin L., Chen P., Li D., Gao W., Dong G. (2022). Colorectal cancer immunotherapy — recent progress and future directions. Cancer Lett.

[43] Raskov H., Orhan A., Christensen J.P., Gögenur I. (2021). Cytotoxic CD8^+^ T cells in cancer and cancer immunotherapy. Br J Cancer.

[44] Wu Y., Yi M., Niu M., Mei Q., Wu K. (2022). Myeloid-derived suppressor cells: an emerging target for anticancer immunotherapy. Mol Cancer.

[45] van Vlerken-Ysla L., Tyurina Y.Y., Kagan V.E., Gabrilovich D.I. (2023). Functional states of myeloid cells in cancer. Cancer Cell.

[46] Ciardiello F., Ciardiello D., Martini G., Napolitano S., Tabernero J., Cervantes A. (2022). Clinical management of metastatic colorectal cancer in the era of precision medicine. CA Cancer J Clin.

[47] Dekker E., Tanis P.J., Vleugels J.L.A., Kasi P.M., Wallace M.B. (2019). Colorectal cancer. Lancet.

[48] Weng J., Li S., Zhu Z. (2022). Exploring immunotherapy in colorectal cancer. J Hematol Oncol.

[49] Davis C., Naci H., Gurpinar E., Poplavska E., Pinto A., Aggarwal A. (2017). Availability of evidence of benefits on overall survival and quality of life of cancer drugs approved by European Medicines Agency: retrospective cohort study of drug approvals 2009-13. BMJ.

[50] Le D.T., Durham J.N., Smith K.N. (2017). Mismatch repair deficiency predicts response of solid tumors to PD-1 blockade. Science.

[51] Rkein A.M., Ozog D.M. (2014). Photodynamic therapy. Dermatol Clin.

[52] Dolmans D.E.J.G.J., Fukumura D., Jain R.K. (2003). Photodynamic therapy for cancer. Nat Rev Cancer.

[53] Yu X., Zhang Z., Yu J., Chen H., Li X. (2018). Self-assembly of a ibuprofen-peptide conjugate to suppress ocular inflammation. Nanomed Nanotechnol Biol Med.

[54] Li X., Lovell J.F., Yoon J., Chen X. (2020). Clinical development and potential of photothermal and photodynamic therapies for cancer. Nat Rev Clin Oncol.

[55] Kommareddy S., Tiwari S.B., Amiji M.M. (2005). Long-circulating polymeric nanovectors for tumor-selective gene delivery. Technol Cancer Res Treat.

[56] Shi Y., van der Meel R., Chen X., Lammers T. (2020). The EPR effect and beyond: strategies to improve tumor targeting and cancer nanomedicine treatment efficacy. Theranostics.

[57] Ikeda-Imafuku M., Wang L.L.W., Rodrigues D., Shaha S., Zhao Z., Mitragotri S. (2022). Strategies to improve the EPR effect: a mechanistic perspective and clinical translation. J Contr Release.

[58] Wang H.X., Zuo Z.Q., Du J.Z. (2016). Surface charge critically affects tumor penetration and therapeutic efficacy of cancer nanomedicines. Nano Today.

[59] Jiang W., Liang M., Lei Q., Li G., Wu S. (2023). The current status of photodynamic therapy in cancer treatment. Cancers.

[60] Deng B., Wang K., Zhang L., Qiu Z., Dong W., Wang W. (2023). Photodynamic therapy for inflammatory and cancerous diseases of the intestines: molecular mechanisms and prospects for application. Int J Biol Sci.

[61] Baskaran R., Lee J., Yang S.G. (2018). Clinical development of photodynamic agents and therapeutic applications. Biomater Res.

