# Supplementary material for: Ce6-GFFY is a novel photosensitizer for colorectal cancer therapy
Source: Genes Dis. 2024 Oct 28;12(2):101441. doi: 10.1016/j.gendis.2024.101441 (PMC11697048; doi:10.1016/j.gendis.2024.101441)
Supplement: Multimedia component 1 [file mmc1.docx]

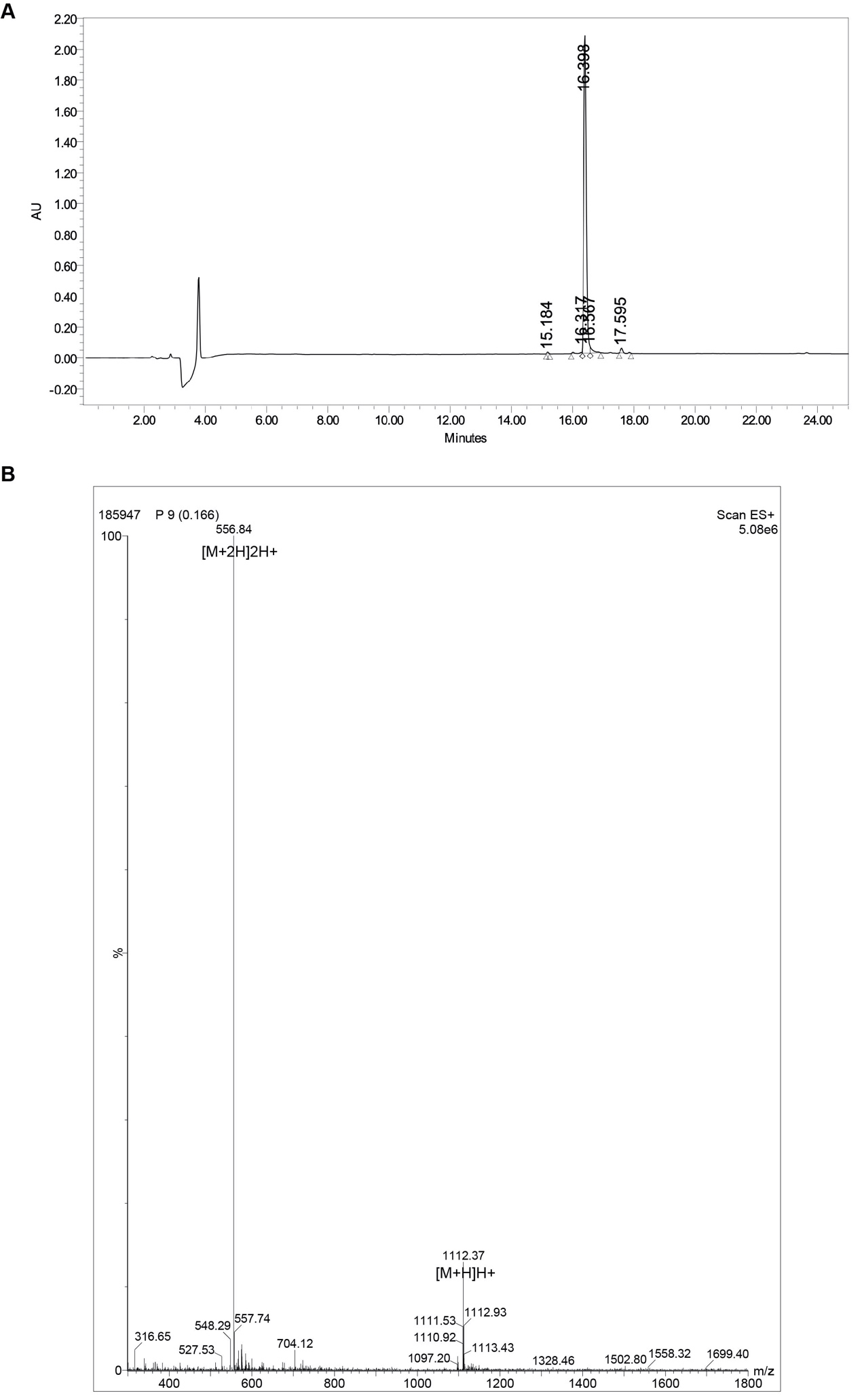


**C**

**
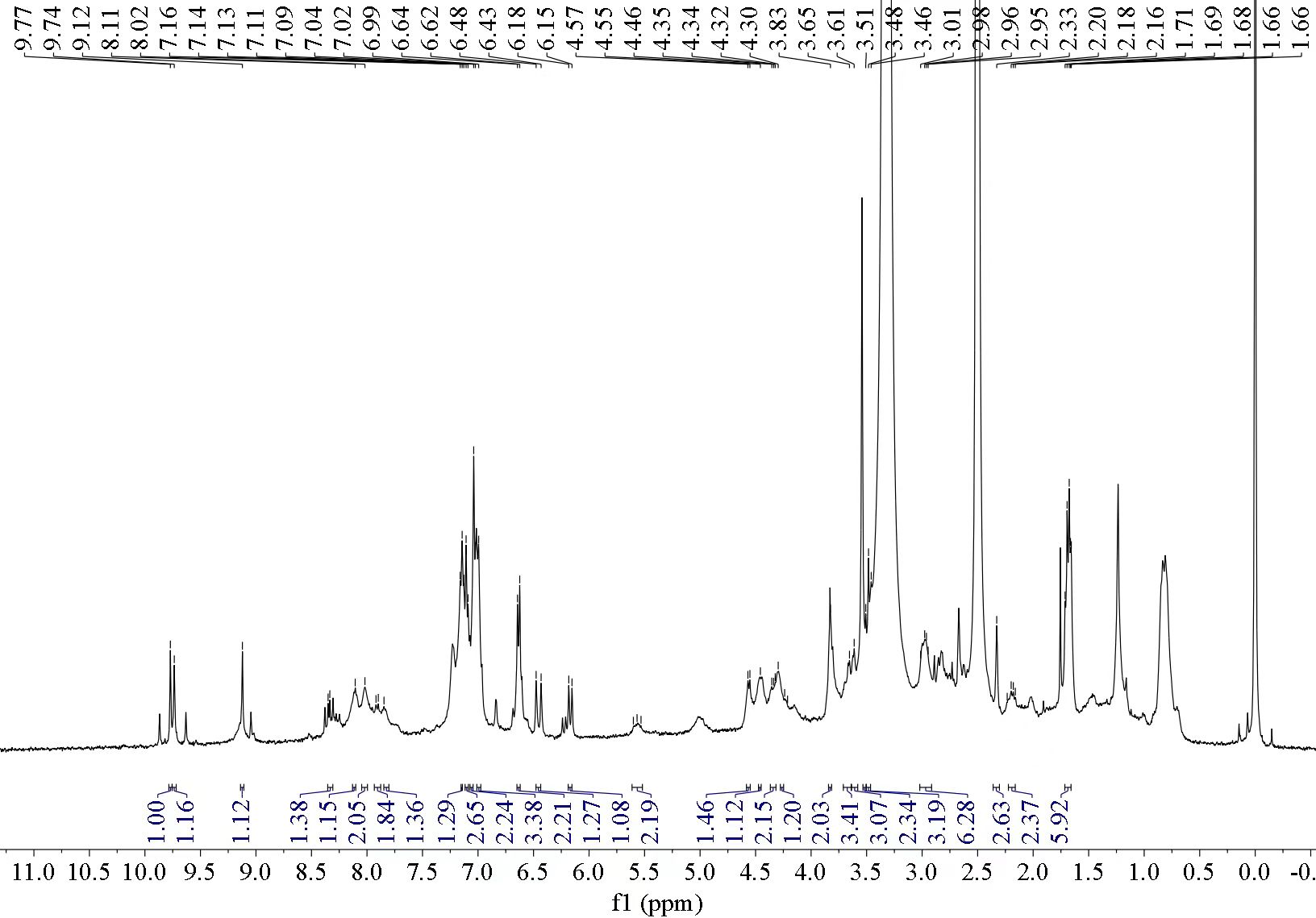
**

**Figure S1**. Characterization of Ce6-GFFY. The HPLC purification **(A)** and MS identification **(B)** of Ce6-GFFY. **(C)** The ^1^H-NMR identification of Ce6-GFFY. MS: calc. M+ =1110.49, obsvd. (M+H) H+ =1112.37. ^1^H NMR (400 MHz, DMSO-*d*_6_) δ 9.77 (s, 1H), 9.74 (s, 1H), 9.12 (s, 1H), 8.34 (d, *J* = 6.2 Hz, 1H), 8.11 (s, 1H), 8.02 (s, 1H), 7.91 (d, *J* = 8.3 Hz, 2H), 7.85 (s, 1H), 7.14 (t, *J* = 6.5 Hz, 1H), 7.10 (d, *J* = 7.0 Hz, 1H), 7.04 (s, 2H), 7.01 (d, *J* = 12.0 Hz, 2H), 6.63 (d, *J* = 8.0 Hz, 3H), 6.45 (d, *J* = 17.9 Hz, 2H), 6.17 (d, *J* = 11.6 Hz, 1H), 5.64 – 5.50 (m, 2H), 4.56 (d, *J* = 7.2 Hz, 1H), 4.46 (s, 1H), 4.39 – 4.30 (m, 2H), 4.27 (d, *J* = 23.4 Hz, 1H), 3.83 (s, 2H), 3.65 (s, 3H), 3.61 (s, 3H), 3.51 (s, 2H), 3.47 (d, *J* = 9.5 Hz, 3H), 2.97 (dd, *J* = 16.8, 10.4 Hz, 6H), 2.33 (s, 2H), 2.20 (d, *J* = 29.3 Hz, 2H), 1.68 (q, *J* = 7.4 Hz, 6H). HPLC, high performance liquid chromatography; MS, mass spectrometry; ^1^H NMR, proton nuclear magnetic resonance.

**
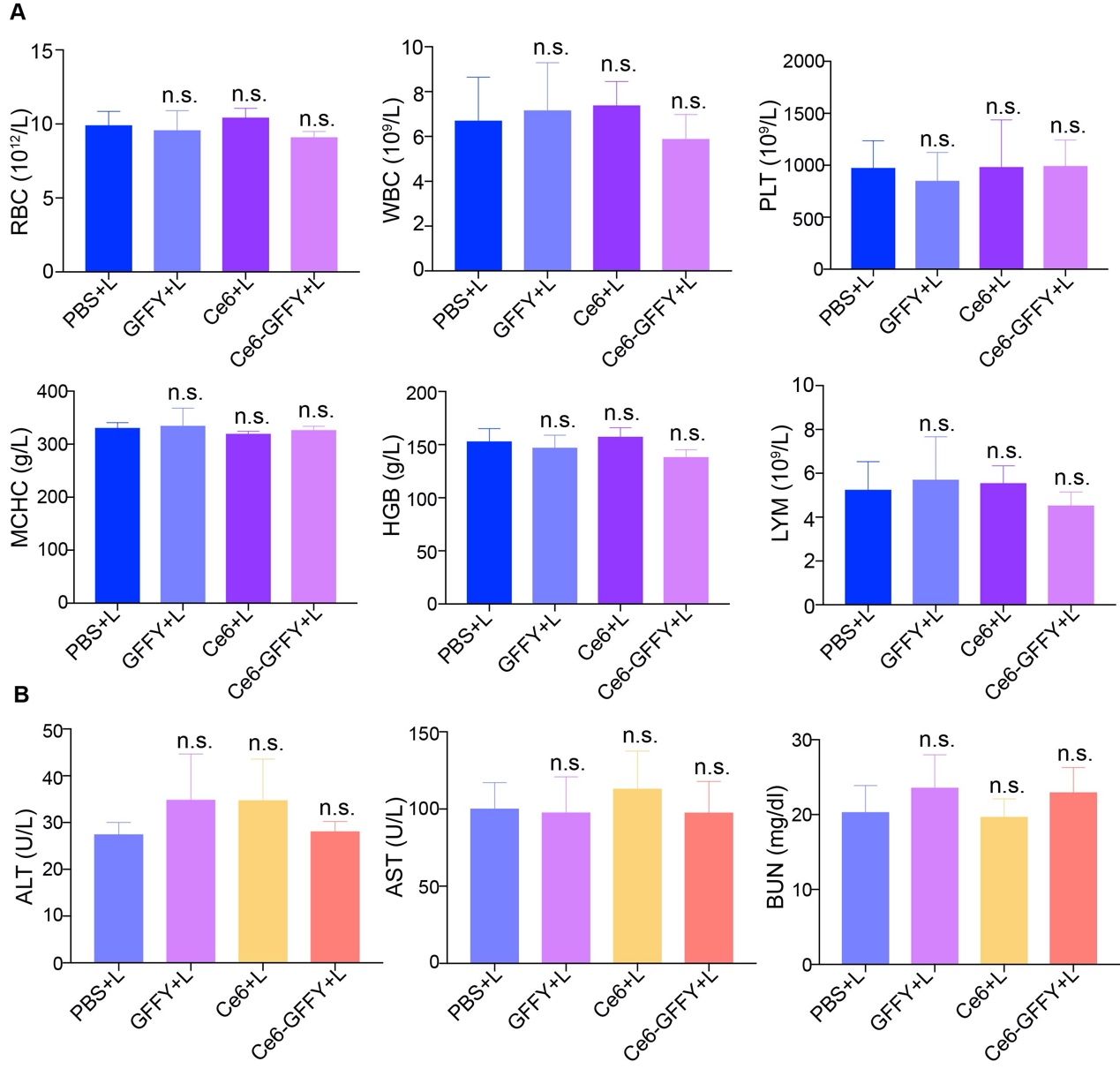
**

**Figure S2**. Ce6-GFFY shows little side-effects. (**A**) Routine blood test of mice treated with PBS, GFFY (2.5 mg/kg), Ce6 (2.5 mg/kg), and Ce6-GFFY (5 mg/kg). n = 5. RBC, red blood cell count; LYM, lymphocyte ratio; WBC, white blood cell count; PTL, blood platelet counts; MCH, Mean corpuscular hemoglobin content; HGB, Haemoglobin concentration. (**B**) Liver and kidney function examination of mice treated with PBS, GFFY (2.5 mg/kg), Ce6 (2.5 mg/kg), and Ce6-GFFY (5 mg/kg). n = 5. AST, Aspartate aminotransferase; ALT, Alanine aminotransferase; BUN, blood urea nitrogen. "L" in "PBS+L", "GFFY+L", "Ce6+L", "Ce6-GFFY+L": laser irradiation. Statistical analyses were performed using one-way ANOVA; Bars, SD; n.s., not significant.
